# Restoring the epigenetic landscape of lung microbiome: potential therapeutic approach for chronic respiratory diseases

**DOI:** 10.1186/s12890-023-02789-7

**Published:** 2024-01-02

**Authors:** Azadeh KavianFar, Hamidreza Taherkhani, Ali Ahmadi, Mahdieh Salimi, Hossein Lanjanian, Ali Masoudi-Nejad

**Affiliations:** 1 Laboratory of Systems Biology and Bioinformatics (LBB), Department of Bioinformatics, Kish International Campus, University of Tehran, Kish Island, Iran; 2 Molecular Biology Research Center, Systems Biology and Poisonings Institute, Tehran, Iran; 3 Department of Medical Genetics, Institute of Medical Biotechnology, National Institute of Genetic Engineering and Biotechnology (NIGEB), Tehran, Iran; 4 Cellular and Molecular Endocrine Research Center, Research Institute for Endocrine Sciences, Shahid Beheshti University of Medical Sciences, Tehran, Iran; 5 Laboratory of Systems Biology and Bioinformatics (LBB), Institute of Biochemistry and Biophysics, University of Tehran, Tehran, Iran

**Keywords:** Chronic respiratory diseases, Pseudomonas, Lung microbiome, Epigenetic

## Abstract

**Background:**

Chronic respiratory diseases, such as chronic obstructive pulmonary disease (COPD) and bronchiectasis, present significant threats to global health. Recent studies have revealed the crucial role of the lung microbiome in the development of these diseases. Pathogens have evolved complex strategies to evade the immune response, with the manipulation of host cellular epigenetic mechanisms playing a pivotal role. There is existing evidence regarding the effects of *Pseudomonas* on epigenetic modifications and their association with pulmonary diseases. Therefore, this study aims to directly assess the connection between *Pseudomonas* abundance and chronic respiratory diseases. We hope that our findings will shed light on the molecular mechanisms behind lung pathogen infections.

**Methods:**

We analyzed data from 366 participants, including individuals with COPD, acute exacerbations of COPD (AECOPD), bronchiectasis, and healthy individuals. Previous studies have given limited attention to the impact of *Pseudomonas* on these groups and their comparison with healthy individuals. Two independent datasets from different ethnic backgrounds were used for external validation. Each dataset separately analyzed bacteria at the genus level.

**Results:**

The study reveals that *Pseudomonas*, a bacterium, was consistently found in high concentrations in all chronic lung disease datasets but it was present in very low abundance in the healthy datasets. This suggests that *Pseudomonas* may influence cellular mechanisms through epigenetics, contributing to the development and progression of chronic respiratory diseases.

**Conclusions:**

This study emphasizes the importance of understanding the relationship between the lung microbiome, epigenetics, and the onset of chronic pulmonary disease. Enhanced recognition of molecular mechanisms and the impact of the microbiome on cellular functions, along with a better understanding of these concepts, can lead to improved diagnosis and treatment.

## Background

The microbiome refers to the collection of microorganisms that live within and on the human body, and it has been shown to have a significant impact on human health and disease [1]. Examining lung diseases from a microbiome perspective is crucial as the human lung is home to a diverse population of microorganisms, collectively known as the lung microbiome [2]. Recent studies have shown that an imbalance in the lung microbiome, known as dysbiosis, can contribute to the development and progression of diseases [3].

By examining the lung microbiome, researchers can identify new targets for therapeutic interventions and gain insight into the complex interactions between the microorganisms, host, and environment that contribute to health and disease [4, 5]. The field of probiotic applications is expanding rapidly, with a focus on their use in managing respiratory tract infections. In a study conducted by Giovanna Batoni and her colleagues, they identified *Lactobacillus acidophilus* as a promising candidate for further investigation in the development of potential treatments for controlling *Pseudomonas aeruginosa* infections [6]. Scientists explored the effectiveness of supplementing patients with COPD using *Lactobacillus casei Shirota*. The findings revealed enhancements in both lung function and quality of life, indicating a possible beneficial role for probiotics in managing COPD [7, 8]. A study by Huang et al. explored the impact of dietary prebiotics, on asthma control. Consumption of prebiotic-rich foods was associated with reduced airway inflammation in asthmatic individuals, highlighting the potential of dietary interventions in asthma management [9].

Fecal microbiota transplantation (FMT) and a high-fiber diet resulted in reduced local and systemic inflammation while also offering protection against alveolar destruction and cellular apoptosis [10]. A deeper understanding of the lung microbiome has the potential to improve our ability to diagnose and treat lung diseases, leading to better outcomes for patients [11]. This is achieved through fecal transplantation from donors whose microbial diversity is well-suited for this purpose. Long Wen et al. found that Pseudomonas aeruginosa leads to a reduction in both the diversity and quantity of gut microbiota in mice with pneumonia, resulting in metabolic imbalances. When fecal microbiota transplantation (FMT) was administered, this situation improved in mice with pneumonia. That helped restore the balance between Treg and Th17 cells, consequently alleviating lung inflammation and injury in mice infected with Pseudomonas aeruginosa pneumonia by regulating gut microbiota and addressing metabolic dysfunction [12].

Epigenetics encompasses heritable changes in gene function that occur independently of alterations in the DNA sequence [13, 14]. Types of epigenetic changes include DNA methylation, histone modification, and miRNA regulation [15–17]. Epigenetics plays a crucial role in the relationship between the microbiome and lung disease [18, 19]. The microbiome in the lungs is involved in regulating various biological processes such as cell proliferation, differentiation, stress response, and pathogenesis. Studies have shown that certain microRNAs are dysregulated in chronic lung diseases such as COPD, IPF, and CF and may contribute to the development and progression of these diseases by regulating inflammation, cell proliferation, tissue remodeling, and the immune response to infection [14, 17, 18, 20].

In the context of the lung microbiome, epigenetic changes can impact the balance of microorganisms within the lung and lead to dysbiosis, which can contribute to the development and progression of lung diseases. The interplay between the microbiome and the epigenome is intricate and mutually influential [13]. A better understanding of the role of epigenetics in the microbiome-lung disease relationship has the potential to provide new insights into the development and progression of lung diseases, as well as new targets for therapeutic interventions [21].

*Pseudomonas* is a type of bacteria that can cause respiratory infections and disease in individuals with weakened immune systems, such as those with cystic fibrosis, chronic obstructive pulmonary disease (COPD), or severe asthma [22]. *Pseudomonas aeruginosa (P. aeruginosa*) infection represents a significant danger to individuals with cystic fibrosis (CF). Within the context of this infection, *P. aeruginosa* typically inhabits the thickened mucus lining the airways. This mucus not only offers a supportive structure and a nutrient-rich habitat for the bacterium to thrive but also rarely comes into direct contact with the underlying airway epithelial cells [23]. The presence of *Pseudomonas* in the respiratory tract can disrupt the composition of the respiratory microbiome, resulting in alterations to its typical functions and an elevated susceptibility to respiratory infections [18, 24]. Studies have also shown that *Pseudomonas* can impact the epigenetic regulation of host cells. These changes in gene expression can contribute to the development and progression of respiratory disease [25].

Aforementioned, the mechanism of pathogens interplay with the lung cells are in the question yet, the role of the epigenetics and the connection of *Pseudomonas* and epigenetic have been mentioned on the previous studies. Our main goal is to directly investigate the effect of the *Pseudomonas* pathogen on the COPD as a chronic lung disease. Thus, we compared the composition of the lung microbiome in healthy, COPD, AECOPD, and bronchiectasis subjects using metagenomic data. We categorized the bacteria with high prevalence in each disease and found that the prevalence of the *Pseudomonas* genus is common in all chronic lung diseases and not in the healthy dataset. Our results beside the previous study could propose the epigenetic changes as the cellular mechanism of *Pseudomonas* infection however this idea needs more investigations.

## Methods

### Data acquisition

We carried out a comprehensive screening process of existing datasets on chronic lung diseases using biological samples. The datasets were then grouped based on the V region of the 16s rRNA gene. The results of the screening process led to the selection of the lung sputum biological sample and the V4 region for further analysis. Additionally, a control dataset in healthy individuals for lung sputum samples was also selected for comparison. We selected a total of 820 samples from 366 patients. All the data used in the study was obtained from EBI databases, a reliable source of biological information. The selection of these datasets and the careful screening process were important in ensuring the validity and accuracy of the results of the study.

### Data preprocessing

The process of preparing the data involved several crucial steps to ensure the accuracy and quality of the results. The first step was demultiplexing, which involved separating the different samples present in the dataset based on the single-end or paired-end sequence information. This step was performed using QIIME2 version 2020.6, a widely used software for microbial analysis [26]. After demultiplexing, the samples were denoised to remove any noise present in the data. The DADA2 technique was utilized for this step, which allowed for an evaluation of the quality of the denoised reads [27]. Finally, the filtered raw sequencing data were subjected to a thorough cleaning process to eliminate any samples with fewer than 5000 reads. This step was taken to ensure that the final results were based on high-quality data and to minimize the potential for errors. In conclusion, the data preparation process was crucial to the success of the study and involved several important steps to ensure the accuracy and reliability of the results.

### Taxonomic analysis

The process of taxonomic analysis in QIIME2 version 2020.6 involves a series of crucial steps to obtain meaningful results. The first step involves the clustering of Operational Taxonomic Units (OTUs) based on their similarity, which is determined by a similarity of over 97%. This can be achieved through the use of two methods, the Denovo and closed reference clustering methods [28]. The next step involves the classification of the 16S rRNA gene sequences using a Scikit-Learn naive Bayes machine-learning classifier that is based on the GreenGenes database.

### Alpha and beta diversity

The data obtained from the above steps is then evaluated using various indices, including the Shannon index, which is used to determine alpha diversity, the weighted UniFrac, which is used to evaluate beta diversity, and the primary coordinate analysis (PCoA).

The taxonomic data is analyzed at the genus level. The relative abundances of each genus in each sample are calculated for each dataset individually. This step helps to provide a comprehensive understanding of the taxonomic composition of the data, which can be useful in various applications, such as environmental monitoring, and disease diagnosis among others. The use of clustering methods, machine-learning classifiers, and various indices helps to provide a comprehensive understanding of the taxonomic composition of the data, which can be useful in various applications.

#### External validation data

In order to corroborate the findings from the preceding stages, we repeated the analyses detailed in the prior step using two distinct datasets: one comprising data from healthy subjects and the other containing data from patients with COPD (refer to Table 1).

**Table 1:** Dataset information

	Access number	Disease	Final samples	Number of patients	Sex (%female)	Age	Platform	Country
Main Data	PRJEB9607	Healthy	101	81	62	58	Single end	Korean
	PRJNA377739	COPD	584	101	42	67	Paired end	British
	PRJNA491749	AECOPD	102	112	37	66	Paired end	Canadian
	PRJEB14304	Bronchiectasis	33	72	68	62	Single end	British
External Validation Data	PRJNA491861	Healthy	122	124	73	61	Paired end	British
	PRJNA299077	COPD	242	87	25	61	Single end	British

## Results

### Data acquisition

As depicted in Table 1, the analysis of 16 s rRNA genes in lung sputum was carried out using five distinct datasets, including patients with pulmonary disease and healthy individuals. In the “Availability of data and materials” section, the link to the data obtained from the EBI database is mentioned. The filtered samples consisted of those whose sequence length was less than 5000.

### Data and taxonomic analysis

**Fig. 1:**
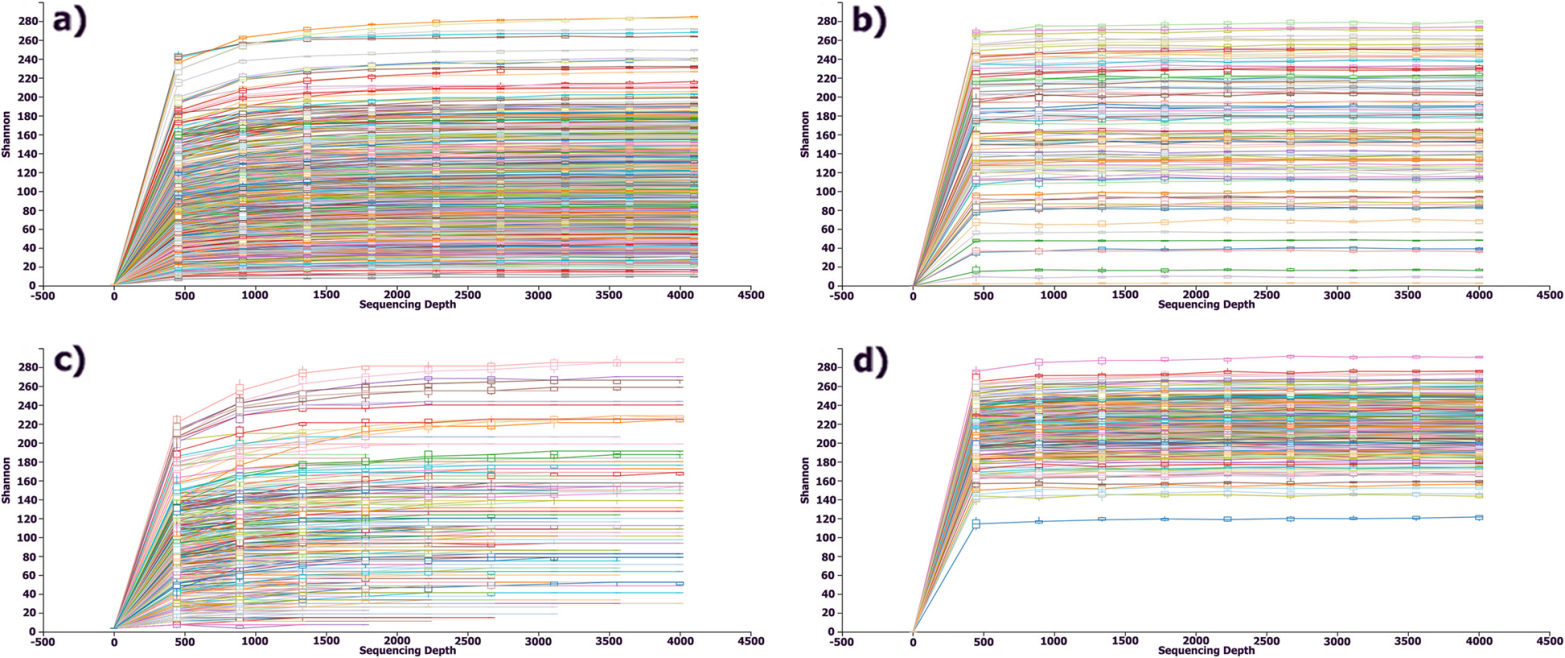
Alpha Rarefication of datasets: **a** COPD; **b** AECOPD; **c** Bronchiectasis; **d** Healthy

**Fig. 2:**
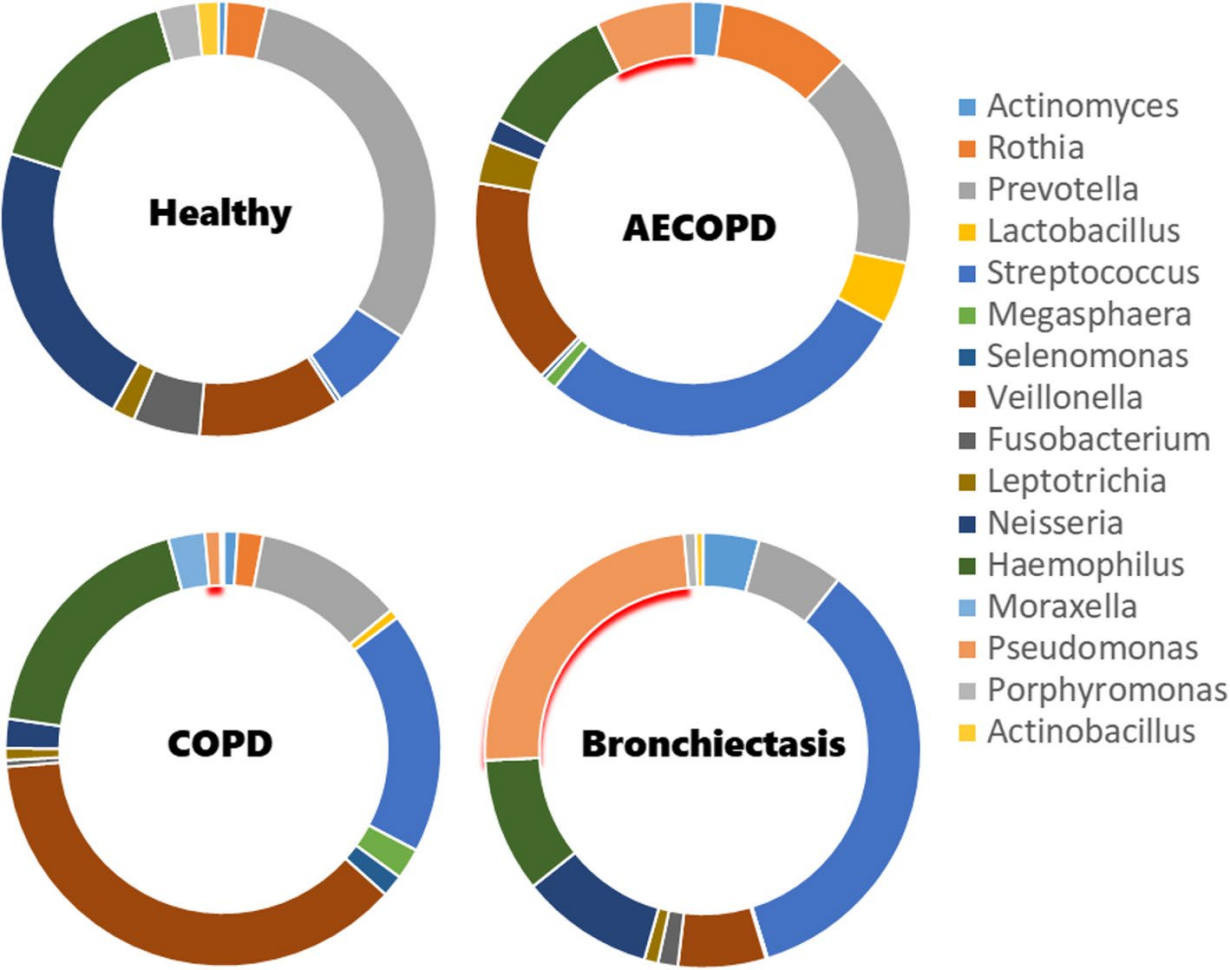
High abundance genera in disease

Alpha-rarefaction separations that were shown in Fig. 1 are done to account for differences in sequencing depth and to ensure that rare species are not missed. Phylogenetically, the diversity of microbial communities was evaluated using four metrics: mean-nearest-taxon-distance (MNTD), nearest-taxon-index (NTI), mean phylogenetic distance (MPD), and net-relatedness index (NRI). The comparison between each sample pair was made using a distance calculation, such as Bray–Curtis or UniFrac, which produced a matrix of pairwise distances. We determined the relative abundance of bacteria at the genus level in three databases for chronic lung diseases, including bronchiectasis, COPD, AECOPD, and a healthy database. The bacteria that showed a high relative abundance in each illness are considered potential candidates. As illustrated in Fig. 2, *Pseudomonas* bacteria (which are highlighted in red) were detected in all patients with chronic lung diseases, whereas it was absent in healthy individuals.

*Rothia* was found to have a significant relative frequency in all datasets except in bronchiectasis. *Actinomyces* was found to have a significant relative frequency in the mentioned diseases, while its relative frequency in the healthy dataset was not significant. *Prevotella, Streptococcus, Veillonella, Neisseria,* and *Haemophilus* were found to have a significant frequency in chronic lung disease and healthy individuals and were considered as “housekeeping genes” in the lung microbiome.

The following genera of bacteria were found to have low abundance only in healthy individuals: *Bacteroides, Butyrivibrio, Peptococcus, Filifactor, Peptostreptococcus, Bulleidia, Cardiobacterium,* and *Mycoplasma*. *Tannerella* and *Catonella* were observed to have low abundance in all datasets except AECOPD. *Porphyromonas* was found to have low abundance in all three disease datasets but was not observed in healthy individuals. *Bifidobacterium* and *Scardovia* were only seen with low abundance in COPD and AECOPD. *Leptotrichia, Ochrobactrum,* and *Actinobacillus* were only seen with low abundance in bronchiectasis. *Acinetobacter* was only seen with low abundance in AECOPD and *Turicibacter, Blautia*, *Oscillospira, Schwartzia*, and *Clostridium* were only seen with low abundance in COPD.

*Leptotrichia* was found to be in significant abundance in COPD and AECOPD and had a lower abundance in the healthy and bronchiectasis datasets. *Lactobacillus* was significantly abundant in COPD and AECOPD but was absent in the healthy and bronchiectasis datasets. It appears that the high abundance of these two bacteria is related to COPD. *Moraxella* was found in COPD but was not present in other datasets. *Actinobacillus* was found to have the highest relative abundance in healthy individuals and low relative abundance in bronchiectasis and was not present in COPD and AECOPD. It appears that its abundance has an inverse relationship with respiratory disease.

In the examination of AECOPD, there was no substantial difference observed in the microbiome between males and females. As the metadata in other datasets lacked information regarding sex, it was impossible to determine the variations in the microbiome based on gender. Our results showed negative correlations based on their relative abundance, which were found between *Pseudomonas* and *Prevotella, Veillonella, Streptococcus*, and *Capnocytophaga* in bronchiectasis whereas there was not any significant correlation between *Pseudomonas* and other genera in chronic lung disease. These findings indicate that the relationship between *Pseudomonas* and other genera may differ among respiratory diseases.

#### External validation data

We conducted the same analyses mentioned in the previous steps on two distinct datasets concerning healthy individuals and patients with COPD (Table 1). In the dataset related to healthy individuals, *Pseudomonas* was not observed, while in the COPD dataset, *Pseudomonas* was observed with a significant frequency.

#### Statistical analysis

In this section, we conducted Chi-Square and Mann–Whitney statistical analyses to assess the presence and abundance of *Pseudomonas* bacteria in various chronic lung diseases compared to the healthy state. Based on the results obtained in Table 2, a significant difference in the abundance of this bacterium was observed between the diseased and healthy states.

**Table 2:** Comparison of present and abundance of *Pseudomonas* between disease and healthy

	Disease	Number of final samples	The presence of *Pseudomonas*		*P*-value Chi-square	Abundant colony of *Pseudomonas*			*P*-value Mann–Whitney
			Positive	Negative		Median	Q1	Q3	
Main Data	Healthy	101	14	87	-----	0	0	0	-----
	COPD	584	168	416	0.00174	0	0	4	0.00001
	AECOPD	102	88	14	0.00001	40.5	10.25	190.75	0.00001
	Bronchiectasis	33	22	11	0.00001	13	0	1630	0.00001
External Validation Data	Healthy	122	18	104	-----	0	0	0	-----
	COPD	242	120	122	0.00001	0	0	173	0.00001

## Discussion

In this study, we conducted a comparison of the respiratory microbiome among individuals with chronic lung diseases, including Chronic Obstructive Pulmonary Disease (COPD), Acute Exacerbation of Chronic Obstructive Pulmonary Disease (AECOPD), non-cystic fibrosis bronchiectasis, and healthy controls. This comparison was based on sputum samples obtained from the V4 area, and we analyzed a total of 820 samples from 366 patients. We found that *Pseudomonas* was present in significant frequencies across all disease datasets, while its abundance was markedly lower in the dataset of healthy individuals. Validation results using independent data have further confirmed our findings. Thus, we are investigating the impact of this pathogen on the epigenetics of chronic lung diseases.

In study by Faure et al., it has been suggested that the presence of *Pseudomonas* is harmless in healthy people [29]. However, our study has explored this issue and found a substantial and distinct difference between healthy individuals and those with COPD, bronchiectasis, and ACOPD. This difference is quite pronounced.

**Fig. 3:**
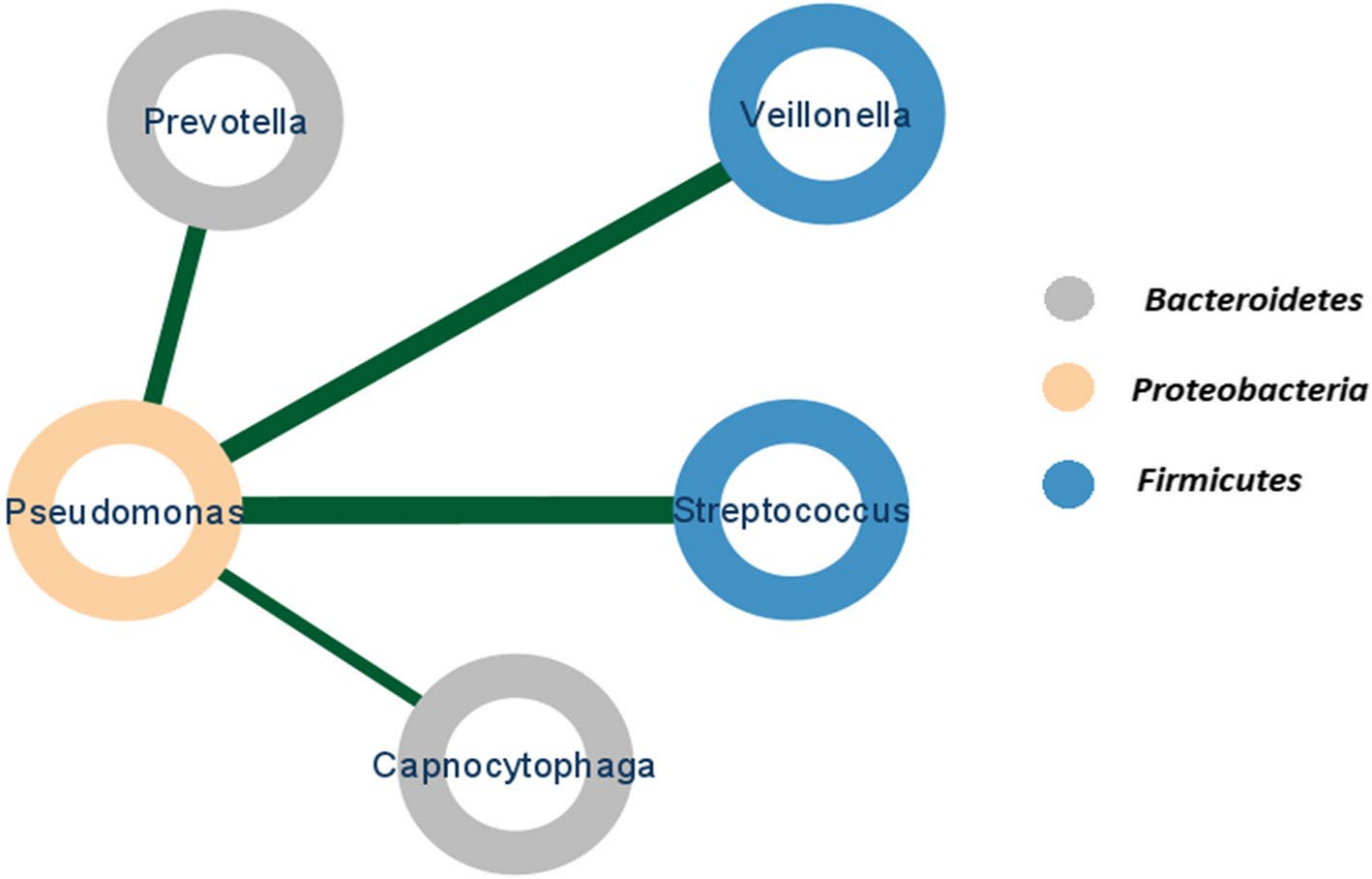
Correlation between *Pseudomonas* and other genera in bronchiectasis based on their relative abundance. The colors of nodes represent bacteria at the phylum level. Blue represents *Firmicutes*, gray represents *Bacteroidetes*, and orange represents *Proteobacteria*. Edges show a negative correlation above 0.4 in bacteria abundance. The thickness of the edges in the network represents the strength of the connections between bacteria

The observed negative correlation between *Pseudomonas* and *Bacteroidetes*/*Firmicutes* in bronchiectasis (Fig. 3) is likely due to their competition for shared resources and the production of antimicrobial compounds that hinder the growth of competing bacterial groups [30].

*Pseudomonas. aeruginosa*, a gram-negative bacterium, which is commonly found in the respiratory tract, can modulate epigenetic mechanisms in host cells to promote its own survival and colonization [31]. Overall, research suggests that *Pseudomonas* may play an important role in shaping the epigenetic landscape of the respiratory microbiome, which can have implications for respiratory health and disease.

DNA methylation is a process by which methyl groups are added to the DNA molecule. This can lead to changes in gene expression by blocking the access of the transcription machinery to the DNA. *Pseudomonas* can modulate host gene expression by changing host ncRNA expression or host DNA methylation pattern in a way that benefits the bacteria [25, 32, 33].

*Pseudomonas* is known to modulate the expression of a wide range of host genes to establish and maintain chronic infections [33]. Some examples of host genes that are affected by *Pseudomonas* include:

- Genes involved in inflammation: *Pseudomonas*’ ability to upregulate pro-inflammatory genes, like TNF-alpha and IL-8, fosters a pro-inflammatory environment conducive to bacterial growth. Furthermore, TNF-alpha and IL-8, known for their roles in infection-related immune responses and their implication in chronic lung diseases such as COPD and bronchiectasis, are suggested to be influenced by the presence of *Pseudomonas* in the respiratory microbiome, potentially affecting the development and progression of chronic lung diseases [34].
- Genes involved in host defense: *Pseudomonas* can downregulate the expression of host defense genes, such as those encoding antimicrobial peptides, to evade the host’s defenses. The downregulation of these genes may result in a weakened immune response and increased susceptibility to infections and inflammation [35]. This can contribute to the progression of the disease and make it more difficult to treat. Genes involved in the innate immune response, such as toll-like receptors (*TLRs*) and nucleotide-binding oligomerization domain-like receptors (*NLRs*), have been implicated in the pathogenesis of chronic obstructive pulmonary disease (COPD) and other respiratory disorders [36]. Similarly, changes in the expression of genes involved in adaptive immune responses, such as *T-cell* receptors and immunoglobulin genes, have been observed in individuals with these conditions [37]. Alterations in host defense genes can result in a weakened immune response, leading to increased susceptibility to infections and inflammation. In other cases, changes in these genes may result in an overactive immune response, leading to tissue damage.
- Genes involved in cell signaling: *Pseudomonas* can modulate the expression of host genes involved in cell signaling pathways, such as those encoding receptor tyrosine kinases, to alter host cell behavior in a manner that favors their survival, enabling them to evade the host’s immune defenses [31–33]. In the context of respiratory diseases, the expression and activation of RTKs may play a crucial role in the development and progression of the disease [38]. Dysregulation of RTK signaling has been implicated in several respiratory diseases, including IPF, Asthma, chronic obstructive pulmonary disease (COPD), and lung cancer.
- Genes involved in apoptosis: *Pseudomonas* can modulate the expression of host genes involved in programmed cell death (apoptosis), to prevent host cells from dying and releasing antimicrobial molecules [39]. Alterations in the expression or activity of genes involved in the apoptosis pathway, such as *Bcl*-2, caspases, and *P53*, have been observed in individuals with chronic obstructive pulmonary disease (COPD), lung cancer, and other respiratory disorders [40]. In some cases, changes in these genes may lead to an impaired ability of the cells in the respiratory system to undergo apoptosis, resulting in increased cell survival and contributing to the development of disease [41]. In other cases, alterations in the apoptosis pathway may result in increased cell death, leading to inflammation and scarring.
- MicroRNA: Research indicates a bidirectional relationship between microbiome composition and host miRNA expression, with potential implications for chronic lung diseases. In COPD, for example, changes in the lung microbiome have been linked to alterations in miRNA expression, which can lead to inflammation and exacerbation of the disease [42]. Additionally, some miRNAs have been shown to directly target bacterial genes, suggesting a direct link between host miRNA expression and microbiome [43]. This highlights the importance of understanding the interplay between the host miRNA expression and the lung microbiome in the development and progression of chronic lung diseases.

The above are examples of host genes affected by *Pseudomonas*, but this bacterium can modulate many other genes depending on the context of infection. Understanding the specific genes and pathways affected by *Pseudomonas* in different types of infections may help to develop new treatments for these infections.

## Conclusion

In this study, we analyzed multiple datasets from individuals with chronic lung diseases and a healthy control group to compare the respiratory microbiome between the two. The results revealed that the genus *Pseudomonas* is abundant and significant in the respiratory microbiome of individuals with chronic lung diseases. Our study also demonstrated that the pathogen *Pseudomonas* can cause epigenetic changes that perpetuate airway inflammation and worsen lung damage. Further investigation is required to fully comprehend the specific mechanisms through which *Pseudomonas* induces these changes and to determine if targeting these changes may offer a promising therapeutic approach for individuals with chronic lung diseases.

## Data Availability

The public datasets analyzed during the current study are available in https://www.ebi.ac.uk/ena/browser/view/PRJNA491749?show=reads https://www.ebi.ac.uk/ena/browser/view/PRJEB14304?show=reads https://www.ebi.ac.uk/ena/browser/view/PRJNA377739?show=reads https://www.ebi.ac.uk/ena/browser/view/PRJEB9607?show=reads https://www.ebi.ac.uk/ena/browser/view/PRJNA491861?show=reads https://www.ebi.ac.uk/ena/browser/view/PRJNA299077?show=reads

## Abbreviations

COPD: Chronic Obstructive Pulmonary Disease
AECOPD: Acute Exacerbation Chronic Obstructive Pulmonary Disease
OTU: Operational Taxonomic Unit
PCoA: Principal Coordinate Analysis
IPF: Idiopathic Pulmonary Fibrosis
CF: Cystic Fibrosis
MNTD: Mean-Nearest-Taxon-Distance
NTI: Nearest-Taxon-Index
MPD: Mean Phylogenetic Distance
NRI: Net-Relatedness Index
*P.** aeruginosa*: *Pseudomonas aeruginosa*
FMT: Fecal microbiota transplantation
ncRNA: Non-coding RNA
